# Draft genome sequence of *Gleimia europaea* DSM 26657

**DOI:** 10.1128/mra.00798-25

**Published:** 2025-11-17

**Authors:** Angela Soghomonian, Mark Soghomonian, Alex Spunde, Chris Daum, Sabine Gronow, Markus Göker, Rekha Seshadri, Kalyani Maitra

**Affiliations:** 1 Department of Chemistry and Biochemistry, California State University 14665, Fresno, California, USA; 2 DOE Joint Genome Institute, Lawrence Berkeley National Laboratory 1666, Berkeley, California, USA; 3 Leibniz Institute DSMZ-German Collection of Microorganisms and Cell Cultures GmbH 28351, Brunswick, Germany; University of Pittsburgh School of Medicine, Pittsburgh, Pennsylvania, USA

**Keywords:** gram-positive bacteria, antibiotic resistance, virulence, opportunist pathogen, soft tissue abscess

## Abstract

Here, we report the draft genome sequence of *Gleimia europaea* DSM 26657, a pathogenic gram-positive bacillus, isolated from a patient with a subcutaneous fistula in 2007 in Germany. The genome is 2.0 Mb in size with 1,813 predicted genes, having only one putative antibiotic resistance gene.

## ANNOUNCEMENT

*Actinomyces* bacteria are pathogenic, gram-positive filamentous bacilli commonly indicated in a variety of polymicrobial soft tissue and oral infections in humans (1, 2). *Actinomyces* is part of the normal human oropharynx, gastrointestinal, and urogenital flora (2). Recently, the opportunistic pathogen *Gleimia europaea* (formerly known as *Actinomyces europaeus*) (3) has been increasingly reported for its role in a variety of infections, including necrotizing fasciitis, soft tissue abscesses, and bacteremia (4–10). *G. europaea* DSM 26657 was first isolated from preoperative wound swabs taken from a patient with a subcutaneous fistula in the right knee joint in 2007 in Germany (11). There is little known about the virulence capabilities of this genus. The full genome sequence of *G. europaea* DSM 26657 will allow for a better understanding of this bacterium’s antibiotic resistance and potential virulence factors.

**Fig 1:**
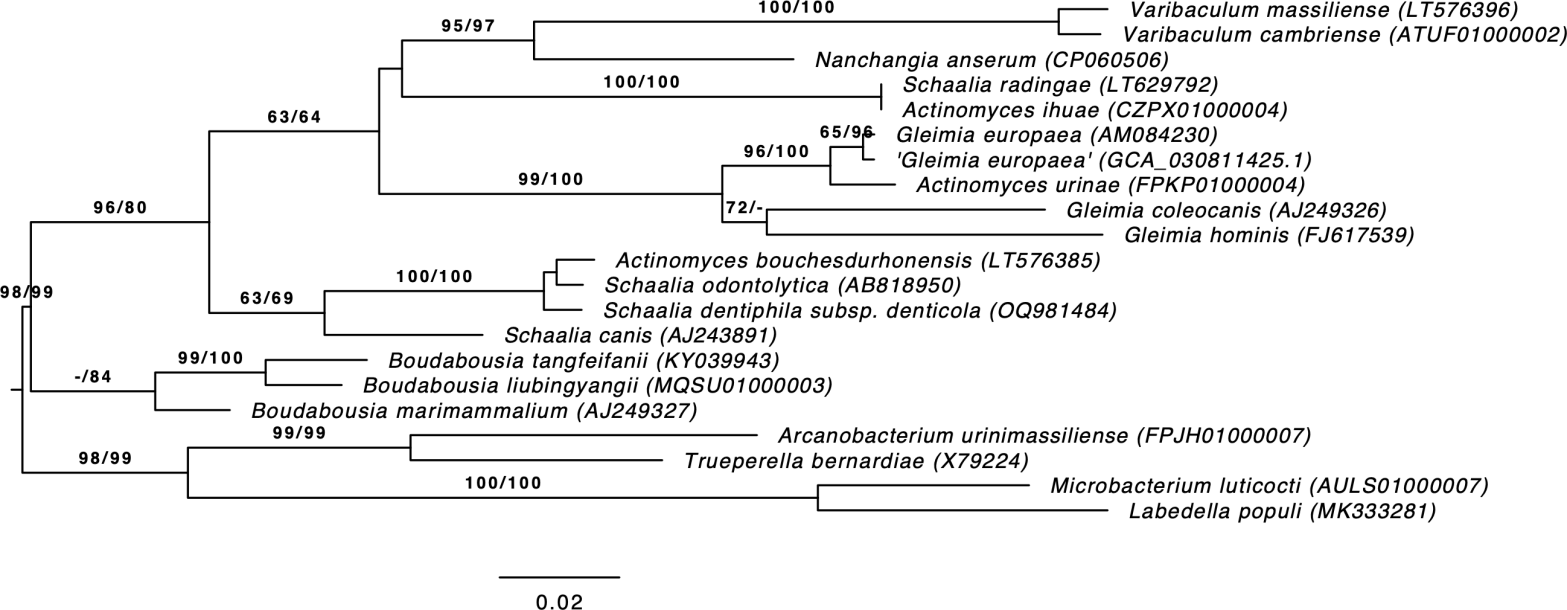
The 16S rRNA-based maximum likelihood tree inferred under the GTR + GAMMA model and rooted by midpoint-rooting using the GGDC server. The numbers above the branches are bootstrap support values when larger than 60% from maximum likelihood (left) and maximum parsimony (right) bootstrapping.

*G. europaea* DSM 26657 was grown in M104 (PYG) supplemented with hemin and vitamin K1 at 37°C under 100% N2 (anaerobic) for 48 h. DNA was prepared using MasterPure Gram Positive DNA Purification Kit Cat. No. MGP04100 (Biosearch Technologies). A ~10 kb library was prepared using Pacific Biosciences’ (PacBio) SMRTbellTM sequencing platform. A total of 1 µg of genomic DNA was sheared around 10 kb using the Megaruptor 3 (Diagenode) or g-TUBE (Covaris) and treated to remove single-stranded ends and repair DNA damage, followed by end-repairing, A-tailing, and ligation with barcoded overhang adapters using SMRTbell Express Template Prep Kit 2.0 (PacBio) and purified with AMPure PB Beads (PacBio). PacBio sequencing primer was then annealed to the SMRTbell template library, and sequencing polymerase was bound to them using Sequel II Binding kit 2.0. The prepared SMRTbell template libraries were then sequenced on a Pacific Biosystems Sequel II sequencer sequencing primer, 8M v1 SMRT cells, and Version 2.0 sequencing chemistry with 1 × 900 min sequencing movie run times. 210,730 filtered subreads were generated, totaling 1,001,084,629 base pairs. Filtered reads of 6,604 kbp were assembled using PacBio’s Hierarchical Genome Assembly Process (HGAP) assembler version smrtlink/8.0.0.80529, HGAP v4 (1.0) with default settings. PacBio read QC, error correction, and adapter trimming were performed by PacBio’s SMRT Link under default conditions. Reads were additionally filtered for the collapsed smartbells (missing adapters) using icecreamfinder.sh (script in the BBTools suite, https://bbmap.org). The final draft assembly was annotated using v.5.0 of the IMG Annotation Pipeline (12) (Table 1). 16S rRNA-based phylogeny was performed using the Genome-to-Genome Distance Calculator (GGDC) [http://ggdc.dsmz.de/] indicating *G. europaea* as the closest relative (Fig. 1) with 99.54% nucleotide identity (13).

**TABLE 1:** Genome features of *G. europaea* DSM 26657

Genome Feature	*G. europaea* DSM 26657
Total scaffold sequence length (bp)	2,020,570
Number of contigs	1
Contig N_50_ (bp)	2,020,570
Average fold coverage (×)	481.6
GC content (%)	56.2
Total genes	1813
Protein coding genes	1744
rRNA genes	9
tRNA genes	49
JGI IMG/M taxon ID	2923563042
NCBI WGS accession number	JAUSQR000000000
NCBI BioProject accession number	PRJNA708468
NCBI SRA accession number	SRR24887453
NCBI BioSample number	SAMN18242993

The genome of *G. europaea* DSM 26657 is predicted to be linear based on the absence of reads overlapping both ends of the contig. Genome annotation revealed the presence of pilus assembly proteins (TadC, CpaF, and TadB); siderophores/iron transport genes (FepC and FepD); an antibiotic efflux pump (MATE); a capsule synthesis protein (CapA); and a predicted class C beta-lactamase (COG1680), suggesting resistance to beta-lactam antibiotics. Ultimately, this high-quality reference genome provides a solid foundation for an array of experiments to gain a thorough understanding of this bacterium’s virulence and antibiotic resistance potential.

## Data Availability

The whole genome shotgun sequencing project for *G. europaea* DSM 26657 is available under NCBI GenBank, accession number JAUSQR000000000. The project data are available under BioProject accession number PRJNA708468, Sequence Read Archive under accession number SRR24887453 and BioSample under accession number SAMN18242993.
